# Prevention of egg contamination by *Salmonella* Enteritidis after oral vaccination of laying hens with *Salmonella* Enteritidis Δ*tolC* and Δ*acrABacrEFmdtABC* mutants

**DOI:** 10.1186/s13567-016-0369-2

**Published:** 2016-08-12

**Authors:** Sofie Kilroy, Ruth Raspoet, Freddy Haesebrouck, Richard Ducatelle, Filip Van Immerseel

**Affiliations:** Department of Pathology, Bacteriology and Avian Diseases, Faculty of Veterinary Medicine, Ghent University, Salisburylaan 133, 9820 Merelbeke, Belgium

## Abstract

Vaccination of laying hens has been successfully used to reduce egg contamination by *Salmonella* Enteritidis, decreasing human salmonellosis cases worldwide. Currently used vaccines for layers are either inactivated vaccines or live attenuated strains produced by mutagenesis. Targeted gene deletion mutants hold promise for future vaccines, because specific bacterial functions can be removed that may improve safety and allow differentiation from field strains. In this study, the efficacy of *Salmonella* Enteritidis *ΔtolC* and *ΔacrABacrEFmdtABC* strains in laying hens as live vaccines was evaluated. The mutants are deficient in either the membrane channel TolC (*ΔtolC*) or the multi-drug efflux systems acrAB, acrEF and mdtABC (*ΔacrABacrEFmdtABC*). These strains have a decreased ability for gut and tissue colonization and are unable to survive in egg white, the latter preventing transmission of the vaccine strains to humans. Two groups of 30 laying hens were orally inoculated at day 1, 6 weeks and 16 weeks of age with 10^8^ cfu of either vaccine strain, while a third group was left unvaccinated. At 24 weeks of age, the birds were intravenously challenged with 5 × 10^7^ cfu *Salmonella* Enteritidis PT4 S1400/94. The vaccine strains were not shed or detected in the gut, internal organs or eggs, 2 weeks after the third vaccination. The strains significantly protected against gut and internal organ colonization, and completely prevented egg contamination by *Salmonella* Enteritidis under the conditions of this study. This indicates that *Salmonella* Enteritidis *ΔtolC* and *ΔacrABacrEFmdtABC* strains might be valuable strains for vaccination of layers against *Salmonella* Enteritidis.

## Introduction

*Salmonella* Enteritidis first emerged in the 1980s as a significant threat to public health worldwide. Eggs were identified as the main food vehicle causing human illness [1, 2]. A sustained commitment of the authorities, implementation of *Salmonella* control programs and serious investment in *Salmonella* research led to international progress in decreasing the incidence of both egg contamination [3] and human infections [4]. Vaccination in particular contributed to the decline in the number of recorded human cases of *Salmonella* Enteritidis [5]. Both inactivated and live vaccines have been shown to reduce *Salmonella* colonization in layers and contamination of eggs [6–8]. Several live vaccines were developed and proven to be efficient against *Salmonella* colonization [9–11]. Live vaccines may stimulate both cell-mediated and humoral immunity, can induce rapid protection by colonization-inhibition and are easy to administer, i.e. through the drinking water [6, 12]. A major concern of live vaccines however is safety, including the possible risk of reversion to virulence [13]. Whole gene deletion mutants are generally considered to be less capable of reversion to a virulent phenotype as compared to strains harboring point mutations or undefined genetic alterations. For *Salmonella* Enteritidis, a lot of knowledge has been generated on the function of many of the chromosomal genes, and targeted deletions of specific genes related to virulence or persistence in a host have been used to construct live vaccine strains [14–18]. In the case of *Salmonella* vaccines for laying hens, the issue of vaccine safety has an additional dimension, as safety should not only include the target species, but also the risk of transmission to humans through consumption of the eggs. Deleting genes important for virulence in mammals, but also deleting genes that are involved in egg white survival can be a key issue because this will prevent transmission of the vaccine strains to the egg consumers.

Egg white survival is a key characteristic of *Salmonella* Enteritidis transmission to humans. Because of the low pH, iron restricting conditions and the presence of a variety of antimicrobial molecules, egg white is an antimicrobial matrix [19]. Lipopolysaccharide (LPS) structure [20], lysozyme inhibitors [21] and protein and DNA damage repair mechanisms [22, 23] are important in egg white survival of *Salmonella*. Deleting genes encoding these functions could thus generate strains with a deficient egg white survival. Recently obtained data suggested that the multi-drug resistance (MDR) pump systems and the TolC outer membrane channel, through which MDR pumps export antibacterial molecules out of the bacterial cell, are also involved in egg white survival [24]. Siderophore export through TolC counteracting iron-deprivation in egg white, or MDR pump-mediated export of antimicrobial molecules out of the bacterial cell may be involved in this [23, 25].

In the current study, we aimed to evaluate the efficiency of the *Salmonella* Enteritidis *ΔtolC* and *ΔacrABacrEFmdtABC* strains, the latter devoid in three MDR efflux pumps, as live vaccines for protection against *Salmonella* Enteritidis egg contamination and tissue colonization in laying hens.

## Materials and methods

### Vaccine and challenge strains

The vaccine strains *ΔtolC* and *ΔacrABacrEFmdtABC* are defined mutants of *Salmonella* Enteritidis 147 phage type 4. The wild type strain 147 was originally isolated from egg white and is resistant to streptomycin. The strain is known to colonize the gut and internal organs to a high level [26, 27]. All mutations were constructed according to the one step inactivation method previously described by Datsenko and Wanner [28]. Briefly, for the *ΔtolC* mutant, a kanamycin resistance cassette, flanked by FRT-sites, was amplified from the pKD4 plasmid with specific primers, homologous with the flanking region of the target gene. The resulting PCR product was used for recombination on the *Salmonella* Enteritidis 147 strain chromosome using the pKD20 helper plasmid encoding the λ Red system, promoting recombination between the native gene and PCR adjusted antibiotic resistance cassette. Recombinant clones were selected on kanamycin containing plates. Replacement of the target gene by the resistance cassette was confirmed by PCR. The deletion was P22-transduced into a new *Salmonella* Enteritidis 147 strain. The antibiotic resistance cassette was eliminated using the pCP20 helper plasmid, encoding the FLP-recombinase, mediating recombination between the FRT-sites flanking the kanamycin resistance cassette. For the *ΔacrABacrEFmdtABC* strain, the procedure was carried out in three steps, successively deleting the *acrAB*, *acrEF* and *mdtABC* genes. P22 transduction was done in the stepwise generated mutants. All targeted genes were completely deleted from start to stop codon, as confirmed by sequencing analysis. *Salmonella* Enteritidis S1400/94 was used as a challenge strain. The characteristics of this strain have been described previously [29].

The challenge and vaccine strains were incubated overnight with gentle agitation at 37 °C in Luria–Bertani (LB) medium (Sigma, ST. Louis, MO, USA). To determine bacterial titers, tenfold dilutions were plated on brilliant green agar (BGA, Oxford, Basingstoke, Hampshire, UK) for the challenge strain. The vaccine strains were plated on LB supplemented with 1% lactose, 1% phenol red and 100 µg/mL streptomycin to determine the titer, because these strains do not grow on traditional *Salmonella* culture media. The vaccine and challenge strains were diluted in HBSS (Hanks Balanced Salt Solution, Invitrogen, Paisley, UK) to 10^8^ cfu/mL.

### Experimental birds

Ninety (90) day-old Lohmann Brown laying hens (De Biest, Kruishoutem, Belgium) were randomly divided into three groups and housed in separate units. Commercial feed and drinking water was provided ad libitum. The animal experiment in this study followed the institutional guidelines for the care and use of laboratory animals and was approved by the Ethical Committee of the Faculty of Veterinary Medicine, Ghent University, Belgium (EC2013/135). Euthanasia was performed with an overdose of sodium pentobarbital in the wing vein.

### Experimental setup

Two different groups (*n* = 30) of animals were orally immunized at day of hatch, at 6 weeks of age and at 16 weeks of age through crop instillation of 0.5 mL containing 10^8^ cfu *Salmonella* Enteritidis 147 *ΔtolC* (group 1) or *Salmonella* Enteritidis 147 *ΔacrABacrEFmdtABC* (group 2). A third group of birds (*n* = 30) was kept as non-immunized but *Salmonella* challenged positive controls (group 3). At the age of 18 weeks, serum samples were taken for quantification of anti-*Salmonella* Enteritidis antibodies in an LPS-ELISA [30]. At the same time, cloacal swabs were taken in each group and bacteriologically analyzed for the presence of the vaccine strains. At 21 weeks of age, all the hens were in lay. Eggs were collected daily during 3 weeks for bacteriological detection of the vaccine strain in the egg content. At 24 weeks of age, all the animals were intravenously inoculated in the wing vein with 0.5 mL containing 5 × 10^7^ cfu of the *Salmonella* Enteritidis challenge strain S1400/94. This protocol was already used previously to produce high levels of internal egg contamination [10, 31]. The eggs were collected daily during 3 weeks after inoculation and analyzed for the presence of the challenge strain. Three weeks after challenge inoculation, all the animals were euthanized by an overdose of pentobarbital in the wing vein. Samples of the spleen, oviduct, ovary, uterus and caecum were aseptically removed for bacteriological quantification of challenge and vaccine strain bacteria.

### ELISA to quantify anti-LPS antibodies

For analysis of anti-*Salmonella* LPS antibodies in serum samples, a previously described indirect ELISA protocol was used [30]. Three 96 well-plates (Sigma) were coated with 100 µL of an LPS solution (10 µg/mL) in 0.05 M carbonate-bicarbonate (pH 9.6; coating buffer) and incubated for 24 h at 4 °C. The LPS was purified from *Salmonella* Enteritidis PT4 strain. The plates were rinsed four times with phosphate buffered saline (PBS, Sigma) supplemented with 0.1% Tween-20 (Sigma; washing buffer) between each step. In the first step, 100 µL PBS (Sigma) supplemented with 1% bovine serum albumin (BSA, Sigma; blocking buffer) was added to the wells for 1 h at 37 °C. The blocking buffer was then removed. Secondly, serum samples of animals from the different groups were diluted in blocking buffer (1:200) and added to the plates (100 µL). As an internal negative control, serum from a *Salmonella* free chick was used. Serum from a chick that had been infected experimentally with *Salmonella* Enteritidis PT4, strain 76Sa88, was used as an internal positive control. The plates were incubated on a shaking platform for 2 h at 37 °C. Thirdly, peroxidase-labelled rabbit anti-chick IgG (100 µL, Sigma) was diluted (1:2000) in blocking buffer and added to the wells for 1 h and 30 min while shaking at 37 °C. Finally 50 µL of TMB substrate (Fisher Scientific, Erembodegem, Belgium) was added to the wells. The reaction was blocked with 50 µL of sulfuric acid (0.5 M). The absorbance was measured in an ELISA reader at 450 nm. Every sample was analyzed in duplicate. Data were shown as S/P ratios, thus [OD(sample) − OD(negative control)]/[OD(positive control) − OD(negative control)]. Negative values were considered as zero.

### Bacteriological examination of the challenged birds

Cloacal swabs taken at week 18 were incubated overnight at 37 °C in buffered peptone water (BPW, Oxoid, Basingstoke, Hampshire, UK). Afterwards a loopful was plated on LB plates supplemented with 1% lactose, 1% phenol red and 100 µg/mL streptomycin (Sigma) for the detection of the vaccine strains *Salmonella* Enteritidis 147 *Δtol*C and *ΔacrABacrEFmdtABC*.

Samples of caecum, spleen, ovary, oviduct and uterus were homogenized in BPW (10% weight/volume suspensions) and tenfold dilutions were made in HBSS (Invitrogen). Six droplets of 20 µL of each dilution were plated on BGA (for quantification of the challenge strain) or on LB supplemented with 1% lactose, 1% phenol red and 100 µg/mL streptomycin (for quantification of the vaccines). After overnight incubation at 37 °C, the number of cfu/g tissue was determined by counting the number of bacterial colonies for the appropriate dilution. Samples that tested negative after direct plating for the challenge strain were enriched in tetrathionate brilliant green broth (Oxoid, Basingstoke, UK) by overnight incubation at 37 °C. After incubation, a loopful of the tetrathionate brilliant green broth was plated on BGA.

### Egg production and bacteriological examination of eggs

Eggs were collected daily for 6 weeks from week 21 onwards and the egg production was determined. Each day, eggs of six hens per group were pooled in one batch, yielding an egg per batch number that varied between one and six. Upon collection, lugol solution and 95% ethanol were used to decontaminate the surface of the eggshell. After decontamination of the eggshell, the eggs were broken aseptically and the total content of the eggs was pooled and homogenized per batch. A volume of 40 mL of BPW was added for each egg to the pooled egg content and incubated for 48 h at 37 °C. To detect the vaccine strains, a loopful of the BPW broth was plated on LB plates supplemented with 1% lactose, 1% phenol red and 100 µg/mL streptomycin. To detect the challenge strain, a loopful of the BPW broth was plated on BGA. Additionally, further enrichment was done overnight at 37 °C in tetrathionate brilliant green broth and after incubation, a loopful of broth culture was streaked onto BGA.

### Statistical analysis

SPSS 22.0 software was used for statistical analysis. Cloacal swabs, batches of eggs and data of cfu *Salmonella*/gram tissue of the caecum, spleen, ovary, oviduct and uterus after enrichment were categorized as either positive or negative. A binary regression model was used to determine differences between the groups. For all tests, differences with p values below 0.05 were considered to be statistically significant.

## Results

### Detection of anti-Salmonella LPS antibodies in serum

**Figure 1:**
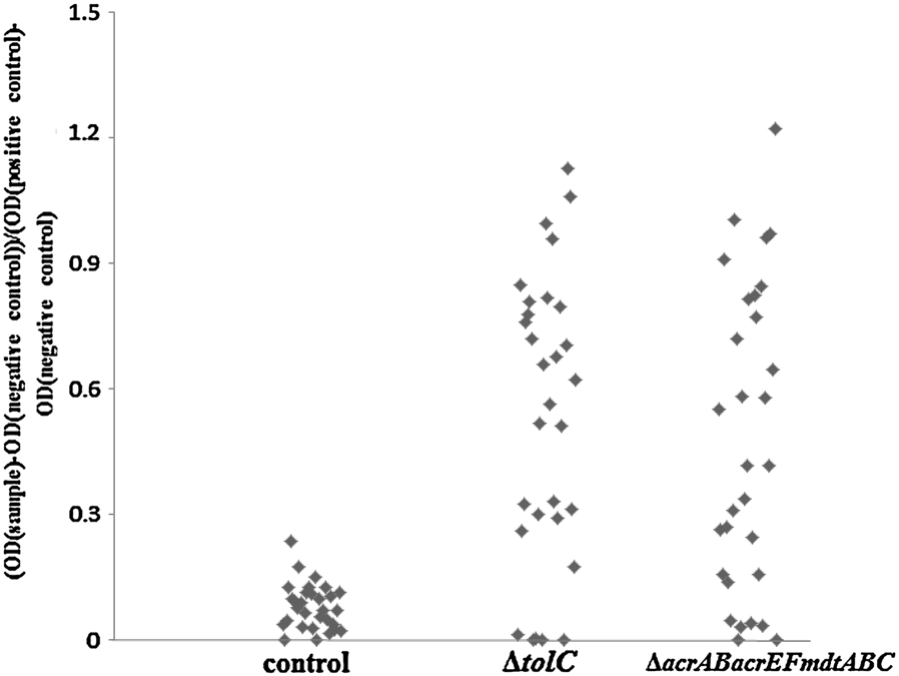
**[OD(sample)** **−** **OD(negative control)]/[OD(positive control)** **−** **OD(negative control)]** **measured in** **the ELISA detecting anti-** ***Salmonella*** **LPS antibodies.** Serum of 18-week old laying hens, vaccinated at day 1, 6 weeks of age and 16 weeks of age with *Salmonella* Enteritidis 147 *ΔtolC* and *Salmonella* Enteritidis 147 *ΔacrABacrEFmdtABC* was analysed.

Data derived from the LPS-ELISA are shown in Figure 1. The data are represented as S/P ratios, thus [OD(sample) − OD(negative control)]/[OD(positive control) − OD(negative control)].

### Analysis of cloacal swabs and eggs for the presence of vaccine strains

Not a single *Salmonella* vaccine isolate was obtained from cloacal swabs or egg content samples.

### Clinical signs and egg production after challenge

Over the whole experiment, there was no reduction in feed and water intake in either of the groups. The egg production rate after infection in the unvaccinated control group dropped to 59% in the first week post-infection (pi) and raised to 75 and 86% in the second and third week pi. The egg production rate did not decrease significantly after challenge in the vaccinated groups. The egg production percentage in the group vaccinated with the *ΔtolC* strain was 60, 100 and 90%, in the first, second and third week after challenge. In the group vaccinated with the *ΔacrABacrEFmdtABC* strain, the egg production percentage was 56, 70 and 68% respectively. Some eggs were thin-shelled and malformed during the first week after infection. At the end of the experiment, 11 hens died in the group of animals vaccinated with the *Salmonella* Enteritidis 147 *ΔacrABacrEFmdtABC* strain because of cannibalism.

### Isolation of the challenge strain from egg contents

Not a single *Salmonella* positive egg batch was detected from animals vaccinated with the *Salmonella* Enteritidis 147 *ΔtolC* and *Salmonella* Enteritidis 147 *ΔacrABacrEFmdtABC* strains (Table 1). During the first week, three egg batches out of 26 were *Salmonella* positive in the non-vaccinated control group at direct plating. In the third week pi, no positive egg batches were found.

**Table 1:** **Percentage of egg content batches positive for the challenge strain** ***Salmonella*** **Enteritidis S1400/94**

Group	Week 1	Week 2
Non-vaccinated	70^a^ (74)^a^	0 (17)^a^
*ΔtolC*	0^b^ (0)^b^	0 (0)^b^
*ΔacrABacrEFmdtABC*	0^b^ (0)^b^	0 (0)^b^

Animals were vaccinated at day one, 6 weeks and 16 weeks of age with 10^8^ cfu of either *Salmonella* Enteritidis 147 *ΔtolC* or *Salmonella* Enteritidis 147 *ΔacrABacrEFmdtABC* strains or kept as non-immunized controls. Results are shown for egg content samples, plated on BGA after BPW (48 h, 37 °C) incubation. Percentage of batches positive after enrichment in tetrathionate brilliant green broth (37 °C, overnight) are shown between brackets. Different superscripts within a column indicate significant differences between the groups (*p* < 0.05).

### Isolation of the challenge strain from the organs at 3 weeks post-infection

No samples were positive at direct plating. Table 2 presents the percentage of *Salmonella*-positive organ samples after enrichment, in vaccinated and non-vaccinated groups, at 3 weeks post challenge. Vaccination with the *Salmonella* Enteritidis 147 *ΔtolC* strain significantly decreased the number of *Salmonella* positive samples in the spleen, caecum and ovary as compared to the control group. Vaccination with the *ΔacrABacrEFmdtABC* strain significantly reduced the number of *Salmonella* positive samples in the ovary and oviduct.

**Table 2:** **Percentage of** ***Salmonella*** **-positive samples after enrichment**

	Control	*ΔtolC*	*ΔacrABacrEFmdtABC*
Uterus	13.3	10	15.9
Spleen	80	50*	63.2
Caecum	30	6.6*	0*
Ovary	70	36.6*	31.6*
Oviduct	46.6	30	5.3*

Samples of uterus, spleen, caecum, ovary and oviduct were taken, 3 weeks post-infection with *Salmonella* Enteritidis S1400/94. Animals were vaccinated at day 1, week 6 and week 16 with either *Salmonella* Enteritidis 147 *ΔtolC* or *Salmonella* Enteritidis 147 *ΔacrABacrEFmdtABC*. Statistically significant differences (*p* < 0.05) in percentage of positive organ samples between vaccinated groups and the non-vaccinated control group are indicated with an asterisk.

## Discussion

Current commercial live vaccines contain strains harboring undefined mutations in one or more genes on the chromosome or defined point mutations. Strains harboring (undefined or defined) point mutations might, however, revert to a virulent phenotype and are thus considered to be unsafe [13, 32]. Future live vaccines should therefore contain fully defined strains carrying (multiple) gene deletions for purposes of safety. Deletion of entire genes additionally permits differentiation from wild type strains, allowing quality control. Numerous experimental vaccines were already tested in various animal hosts, including chickens, but data on the protection of these live vaccines against egg contamination are scarce [10, 17, 18].

Successful attenuation of the wild type strain requires prior knowledge of the pathogen’s virulence factors. A vaccine strain used for the prevention of (vertical) egg contamination of *Salmonella* Enteritidis ideally colonizes and induces local immunity in the reproductive tract. From a public health point of view, it may not persist here and preferably does not survive in egg white. A logical approach is to eliminate genes playing a role in egg white survival. In the current study defined mutants in MDR transporters and the TolC outer membrane channel were used as vaccine strains. The TolC promoter is activated after contact with egg white at 42 °C, but not under standard “in vitro” culture conditions [24]. The TolC outer membrane channel is used by MDR transporters (e.g. *acrAB, acrEF, mdtABC*) to export host antibacterial compounds and bacterial molecules such as siderophores, and is involved in survival in harmful environments, including egg white [33]. The *ΔtolC* and Δ*acrABacrEFmdtABC* vaccine strains can no longer survive in egg white, thereby eliminating the risk of human exposure through eggs [24]. To our knowledge, these genes were never associated with protective immunity in chickens, allowing wild type-like antigen presentation.

The actual immune mechanism explaining the protection against *Salmonella* Enteritidis colonization observed in the current trial is not completely clear. Immunization with *Salmonella* vaccines can induce variable humoral and cell-mediated responses that do not always correlate with acquired resistance to re-infection [34]. A role for humoral responses in the clearance of *Salmonella* infections has been shown for using inactivated vaccines, which are less able to induce cellular responses but are still partially protective [35]. Cell-mediated immunity was not investigated during this trial but for *Salmonella* in poultry, susceptibility to the infection is correlated with a fall in CD4^+^ and CD8^+^ T-lymphocytes and γδ T-lymphocytes in the oviduct, and with T-lymphocyte hyporesponsiveness [36]. Live vaccines have been shown to increase numbers of CD4^+^ and CD8^+^ T-lymphocytes to a certain level in the gut wall [37]. Future studies should further investigate the role of the humoral and cellular immune responses during vaccine-induced protection. Possibly a combination of cell-mediated immunity and a strong humoral response are yielding additional protective effects.

To conclude, data from this trial indicate that *Salmonella* Enteritidis *ΔtolC* and *ΔacrABacrEFmdtABC* strains are safe vaccines that can induce protection against internal organ colonization after intravenous inoculation of a *Salmonella* Enteritidis challenge strain. The vaccine strains were able to completely prevent egg contamination with *Salmonella* Enteritidis in the current in vivo trial.
